# Individual and regional differences in the effects of school racial segregation on Black students’ health

**DOI:** 10.1016/j.ssmph.2024.101681

**Published:** 2024-05-20

**Authors:** Gabriel L. Schwartz, Guangyi Wang, Min Hee Kim, M. Maria Glymour, Justin S. White, Daniel Collin, Rita Hamad

**Affiliations:** a Philip R. Lee Institute for Health Policy Studies, University of California San Francisco, 490 Illinois Street, 7th Floor, San Francisco, CA, 94158, USA; b Urban Health Collaborative & Department of Health Management & Policy, Drexel University Dornsife School of Public Health, 3600 Market St, Philadelphia, PA, 19147, USA; c Department of Epidemiology & Biostatistics, University of California San Francisco, 550 16th Street, 2nd Floor, San Francisco, CA, 94158, USA; d Department of Epidemiology, Boston University School of Public Health, 715 Albany Street, Boston, MA, 02118, USA; e Department of Health Law, Policy & Management, Boston University School of Public Health, 715 Albany Street, Boston, MA, 02118, USA; f Department of Social & Behavioral Sciences, Harvard T.H. Chan School of Public Health, 677 Huntington Avenue, Boston, MA, 02115, USA

**Keywords:** Social determinants of health, Segregation, Schools, Structural racism, Social epidemiology, United States

## Abstract

**Background:**

School racial segregation in the US has risen steadily since the 1990s, propelled by Supreme Court decisions rolling back the legacy of *Brown v. Board*. Quasi-experimental research has shown this resegregation harms Black students' health. However, whether individual or family characteristics (e.g., higher family incomes) are protective against segregation's health harms—or whether segregation is more damaging in regions of the US with fewer public sector investments—remains unclear. We leverage the quasi-random timing of school districts being released from *Brown*-era integration plans to examine heterogeneity in the association between resegregation and Black students' health.

**Methods & findings:**

We took an instrumental variables approach, using the timing of integration order releases as an instrument for school segregation and analyzing a pre-specified list of theoretically-motivated modifiers in the Panel Study of Income Dynamics. In sensitivity analyses, we fit OLS models that directly adjusted for relevant covariates. Results suggest resegregation may have been particularly harmful in the South, where districts resegregated more quickly after order releases. We find little evidence that the effects of school segregation differed across family income, gender, or age.

**Conclusion:**

The end of court-ordered integration threatens the health of Black communities—especially in the US South. Modestly higher incomes do not appear protective against school segregation's harms. Research using larger samples and alternative measures of school segregation—e.g., between districts, instead of within districts—may further our understanding of segregation's health effects, especially in Northern states.

## Highlights

• Past research suggests school segregation harms Black US Americans' health.
• We examine potential effect heterogeneity by region, household income, gender, age.
• Using IV models, we find stronger evidence for health effects in Southern states.
• Little evidence that modestly higher family incomes protected against health harms.
• Larger samples and richer segregation measures are needed to study all regions.

## 1 Introduction

Mounting evidence identifies school racial segregation as a driver of health inequity (Bifulco, Lopoo, & Oh, 2015; Johnson, 2011; Kim et al., 2022; Liu, Linkletter, Loucks, Glymour, & Buka, 2012; Shen, 2018; Wang et al., 2022). For much of US history, Black children were barred from attending school with White children, and Black-only schools were barely funded (Johnson, 2019). This began to change following Supreme Court decisions—most notably *Brown v. Board of Education*—that found school racial segregation unconstitutional (Reardon, Grewal, Kalogrides, & Greenberg, 2012a). Resulting lawsuits throughout the 1960s–1970s put more than 1000 segregated districts under court oversight to enforce integration (Reardon et al., 2012a). This occurred in Southern states, where school segregation was legally explicit, as well as Northern states, where school segregation was more often indirect (e.g., via residential segregation enforced by redlining, housing discrimination, and racist violence) (Joffe-Walt, 2020; Reardon & Owens, 2014; Rothstein, 2017). Court-imposed integration increased Black Americans’ educational attainment and earnings and reduced their rates of incarceration, teenage pregnancy, and self-reported poor health, increasing life expectancy (Hahn, 2022; Johnson, 2011, 2019; Liu et al., 2012).

In its 1991 *Board of Education v. Dowell* decision, however, the Supreme Court shifted gears, ruling that *Brown*-era integration orders were never meant to be permanent (Lutz, 2011). *Dowell* and subsequent decisions allowed districts to obtain releases from integration orders with nominal effort. Nearly half of districts ever under an integration order were subsequently released (Reardon et al., 2012a; Reardon & Owens, 2014). These released districts have steadily resegregated; the number of highly segregated schools with <10% White enrollment has accordingly tripled, today comprising 1 in 5 public schools (Lutz, 2011; Orfield, Ee, Frankenberg, & Siegel-Hawley, 2016; Reardon et al., 2012a; Reardon & Owens, 2014; Reardon & Yun, 2002). Recent research suggests this contemporary school resegregation has increased Black Americans’ preterm birth rates (Shen, 2018), worsened behavioral problems and alcohol consumption among Black youth (Wang et al., 2022), and led to poorer self-rated health and higher rates of binge drinking among Black adults (Kim et al., 2022).

Still unknown is whether the impact of school segregation on health varies across geographic regions, despite known differences across regions' (historical and contemporary) segregation patterns and school financing policies (Johnson, 2019; Lutz, 2011). Existing research suggests integration order releases led to larger school segregation resurgences in the South than the North (Reardon et al., 2012a). But the *consequences* of increasing segregation may also differ across regions. Southern residents face elevated premature mortality (Dollar et al., 2020), lower economic mobility (Connor & Storper, 2020), higher rates of poverty (Artiga, 2016), and state governments that have declined to expand Medicaid under the Affordable Care Act (Artiga, 2016; Jones, 2019), among other barriers to well-being (Parcha et al., 2021). Southern states also suffer more extreme restrictions on Black Americans’ voting rights (Álvarez, 2022; Brockell, 2021; Brennan Center for Justice, 2022) and on the ability of local governments to pass health-promoting legislation (Blair, Cooper, Wolfe, & Worker, 2020). Together, this may mean that Black people whose educational opportunities are curtailed in the South by segregation may have greater difficulty acquiring necessary resources for staying healthy, including through political mobilization.

Similarly, exposure may vary by individual characteristics—such as age, gender, or income. For example, older children, whose development and well-being is more reliant on peer relationships, may be more vulnerable to shocks to their schooling and social environments engendered by increasing segregation (Fuhrmann, Knoll, & Blakemore, 2015; Schmidt, Glymour, & Osypuk, 2017). Conversely, children who are younger when their districts resegregate are exposed to more segregated schooling environments for longer (Johnson, 2011, 2019). Younger children also experience higher levels of segregation, as elementary schools are more segregated than older grades (Reardon et al., 2012a). For gender, school segregation studies indicate girls' health may be more heavily impacted than boys' while in school (Wang et al., 2022). Yet in other studies radically changing young people's social environments—such as the Moving to Opportunity study, which gave vouchers to families in public housing to move into low-poverty neighborhoods—older boys exhibited worse mental health in the long term, even when assigned a theoretically beneficial treatment (Chetty, Hendren, & Katz, 2016; Schmidt et al., 2017). These studies suggest boys may exhibit higher social network vulnerability and struggle to reestablish social ties after a move or social shift—e.g., school district integration plans lapsing. Finally, higher-income families who can afford supplementary educational opportunities for their children may be less impacted by school segregation (Greg & Richard, 2016). Still, evidence on segregation's potential effect heterogeneity remains limited.

In this paper, we analyze variation in the association between school segregation and Black Americans’ health, used the timing of integration order releases as a natural experiment. Examining a pre-specified set of theoretically-motivated modifiers, we evaluated heterogeneity by region as well as by age at first exposure, gender, and childhood household income.

## 2 Methods

### 2.1 Data

We build on two prior studies led by Wang (Wang et al., 2022) and Kim (Kim et al., 2022), which estimated effects of school segregation on children's and adults' health, respectively. Like them, we analyzed data from the Panel Study of Income Dynamics (PSID), the longest-running study of American life. Begun in 1968, PSID recruited a nationally representative sample of largely Black and White households, surveying participants on demographic, socioeconomic, and health topics. PSID then followed families and their descendants annually through 1997 and biennially thereafter.

We linked the PSID with previously-compiled school segregation data (ProPublica, 2014; Reardon, Grewal, Kalogrides, & Greenberg, 2012b): after creating a crosswalk between Census blocks and school districts in ArcGIS (a geospatial analysis software), we merged district data to the PSID based on participants’ residential Census blocks.

For child health, we analyzed outcomes while participants were in school from PSID's Child Development Supplement (1997–2014), a detailed sub-study of PSID children. All waves with available outcome data were analyzed, with some children observed multiple times. The analytic sample included 1248 Black youth contributing 1872 observations.

For adult health, we analyzed outcomes from adult PSID participants who were in school for at least one year after 1991—i.e., during the post-*Dowell* era—with the last adult outcome data contributed in 2017. The analytic sample included 1053 Black adults contributing 4723 observations.

Covariate and exposure missingness was low, ≤3% for all covariates; it is therefore unlikely that imputation of these variables would meaningfully alter estimates or conclusions. Sample sizes did vary, however, across outcomes (see figure notes), largely due to differences in who was asked different questions; e.g., some were only asked of heads of household, only asked in certain waves, or only asked for children at particular ages. To assess outcome missingness, we considered observations where participants were eligible to be asked (Appendix F). Among adults, missingness for outcomes largely varied between <1 and 3%, excepting hours of vigorous physical activity (11%); among children, outcome missingness was moderate (∼10%).

For sample selection flowcharts, please see Appendix G.

### 2.2 Exposures

Our endogenous exposure was within-district school segregation, measured using the Black-White dissimilarity index (DI) (Johnson, 2011; Kim et al., 2022; Lutz, 2011; Orfield et al., 2016; Wang et al., 2022). Ranging from 0 to 1, the widely-used DI represents the proportion of Black (or White) students who would have to change schools to achieve a uniform racial distribution across a district's schools (Massey & Denton, 1988). For example, a value of 0.6 indicates that 60% of Black/White students would have to change schools for a district to have the same percentage of Black and White students in each school (Orfield et al., 2016). For the child health sample (as in Wang, to aid cross-study comparisons), school segregation was operationalized as the mean DI value in children's school districts between their first observed schooling year (“baseline”) and the year in which a given outcome was measured. For the adult health sample (as in Kim), segregation was operationalized as the mean level of school segregation across all observations during participants' schooling years after 1991. These were standardized to aid interpretability.

Importantly, the dissimilarity index is distinct from school composition or the exposure or isolation indices (which are also linked to health, albeit inconsistently (Cohen, Ozer, Rehkopf, & Abrams, 2021; Dudovitz et al., 2021; DuPont-Reyes & Villatoro, 2019)). For example, it is possible to have high or low exposure to Black or White students in either an integrated or segregated district, since the distribution of Black or White students across schools within a district (dissimilarity) is distinct from the racial composition of the district overall. Both segregation and exposure are important objects of study; we focused on the former.

We note, however, that the isolation index (in this case measuring the probability that Black students only attend school with other Black students) and the dissimilarity index are highly correlated. Sensitivity analyses by Kim and Wang in these data show that results when using the dissimilarity vs. isolation indices were functionally identical. For the sake of simplicity and clarity, we thus focus on the dissimilarity index, which also most aligns with our object of study: segregation induced by judicial racial integration orders. These orders specifically required districts to move children between schools to achieve a more equal distribution of racial/ethnic groups (i.e., to lower dissimilarity, though judges did not use that term). Dissimilarity is thus what was targeted by our instrumental variable. Still, we conduct sensitivity analyses assessing whether our core results hold when using the isolation index.

### 2.3 Outcomes

We analyzed an array of child and adult health measures linked to school segregation or school resources, per prior literature (Table 1). School segregation is hypothesized to cause these outcomes through multiple paths, including via fewer school resources during childhood—potentially impacting school nutrition and physical activity opportunities; heightened rates of discriminatory school discipline; subsequent increased stress (including unhealthy coping mechanisms, such as smoking or alcohol consumption); worsened poverty and lower educational quality or attainment affecting adult socioeconomic status; and via social networks, though potential peer effects are complex (Kim et al., 2022; Schwartz et al., 2023). Outcomes included self-rated health (Johnson, 2011), smoking (Walsemann & Bell, 2010), alcohol consumption (Schwartz et al., 2023), mental/emotional/behavioral health (DuPont-Reyes & Villatoro, 2019), physical activity (Mahmood, Sanchez-Vaznaugh, Matsuzaki, & Sánchez, 2022), and physical health diagnoses. We coded these identically to prior work to facilitate direct comparisons with our estimates (Kim et al., 2022; Wang et al., 2022).

**Table 1:** Health outcomes, by stage in the life course.

Outcomes	Child Health	Adult Health
Self-rated health	Poor, fair, or good vs. very good or excellent (binary)	Poor or fair vs. good, very good, or excellent (binary)
Smoking	Ever smoked (binary), ever smoked regularly (binary), number of days smoked in the past month (continuous)	Current smoking status (binary), number of cigarettes per day (continuous)
Alcohol	Ever drank (binary), drank at least monthly (binary), heavy drinking at least monthly (binary; 5+ drinks in one sitting)^a^	Ever drank (binary), heavy drinking (binary; whether drank 3+ drinks per day)^a^
Behavioral health	Diagnosis with a mental/emotional health problem (binary); Behavioral Problem Inventory (continuous scale, 0–27)	Kessler-6 Psychological Distress Scale (continuous scale, 0–24)
Physical activity	Whether students got >30 min of vigorous physical activity for 3+ days per week while in school (binary) and out of school (binary)	Hours of weekly vigorous physical activity (continuous)
Body size	Childhood obesity (binary, body mass index ≥95th percentile)	Body mass index (continuous)
Physical health diagnoses	Asthma (binary)	Heart disease, hypertension, diabetes (all binary)

a We refer to this as “heavy drinking” because binge drinking is officially defined as consuming ≥5 (for men) or ≥4 (for women) alcoholic drinks over a single 2-hour period (National Institute on Alcohol Abuse and Alcoholism, 2020). Unfortunately, PSID did not consistently ask questions about alcohol consumption aligned with this definition. Our coding represents proxies for official measures.

For alcohol and smoking, we examined outcomes across the usage continuum, including both whether respondents smoked/drank and how often and how intensely. This multiplicity of measures is useful in the context of racial health inequities, since inequities are not consistent across the usage continuum nor across the life course: Black teenagers, for example, smoke and drink less than White teenagers on average (El-Toukhy, Sabado, & Choi, 2016; Terry-McElrath & Patrick, 2020), but Black adults suffer higher rates of smoking-attributable mortality (Rostron et al., 2022) and alcohol dependence (Mulia, Ye, Greenfield, & Zemore, 2009).

### 2.4 Covariates

Covariates represented potential confounders or hypothesized modifiers, with “baseline” referring to participants’ first childhood observation. These included binary sex (male vs. female, as a coarse proxy for gender oppression exposures) (Krieger, 2003), baseline childhood household income, baseline parental marital status, birth year fixed effects, state fixed effects, and district demographics (total enrollment, racial composition [% non-Hispanic Black, Hispanic, and non-Hispanic White], proportion of students eligible for free or reduced-price lunch, and residential segregation [measured by the Black-White DI calculated across Census tracts within school districts]). For child health analyses, we additionally adjusted for age at outcome measurement.

### 2.5 Analysis: instrumental variables

As in prior studies (Kim et al., 2022; Wang et al., 2022), we used an instrumental variables (IV) design, a quasi-experimental method increasingly adopted by epidemiologists to reduce the threat of confounding (Glymour, 2006). Briefly, IV designs find some (conditionally) random variable that changes exposure levels but does not otherwise affect the outcome. This random perturbation in the exposure is used to estimate an exposure's effects. Here, we use the timing of integration order releases as an instrument for school segregation. We used a two-stage least squares approach: first, regressing school segregation on order release timing, and second, regressing outcomes on predicted changes to school segregation (derived from the first stage).

For IV estimates to be interpreted causally, several assumptions must hold: (1) the IV must cause the exposure; (2) the IV must not cause the outcome through any path other than through the exposure; and (3) there must be no IV-outcome confounding (Lousdal, 2018). In this case, past sociological research (Lutz, 2011; Orfield et al., 2016; Reardon et al., 2012a) shows that the *timing* of releases was effectively random (conditioning on district size, region, and racial composition), largely depending on bureaucratic fluctuations in arbitrary factors such as the size of different judges’ dockets. Falsification tests in the PSID sample have supported these assumptions in the context of health research (Kim et al., 2022; Wang et al., 2022): districts that did vs. did not experience releases were similar prior to releases, and IV analyses on placebo health outcomes that should not have been affected by changes in school segregation yielded null results.

For child health, we operationalized the IV as the number of years elapsed since a child's district was released from a court order, averaged across all their childhood observations between the start of schooling and the year a health outcome was measured. For adult health, we operationalized the IV as the proportion of participants' school-aged observations after 1991 in which a child attended a released district. In both cases, we limited our samples to districts that were ever under an integration order, as other districts likely differed in important ways. These operationalizations mirror past work, to aid cross-study comparisons (Kim et al., 2022; Wang et al., 2022).

Unlike prior investigations using this quasi-experimental design, however, we allowed both the relation between the instrument and the exposure and between predicted changes in the exposure and the outcome to vary by individual and contextual factors (See Appendix A for equations.). Separate models were estimated for each outcome. We fit stratified models for each value of categorical modifiers, as well as interaction models to formally compare stratified models. All models predicting binary outcomes were estimated as linear probability models, as logistic regression models failed to converge; effect estimates are interpretable as risk differences. Since Wang examined differences by age and gender, we examined only income and region as modifiers of the relationship between school segregation and child health.

### 2.6 Modifiers

We tested whether relationships varied by four pre-specified modifiers: region, gender, childhood household income, and age at initial exposure. Region was defined as within the Census' Southern region (the South) vs. otherwise (the “North”). Gender was proxied by binary sex due to limitations of PSID's gender data (male and female, proxying for men/boys and women/girls). Income was defined as inflation-adjusted baseline household income in the earliest observed year of schooling and dichotomized as below vs. above the median for Black respondents (<$7450 annually *per capita* for adult health analysis; <$32,683 *total* household income for child health analysis). (These operationalizations mirrored past research (Kim et al., 2022; Wang et al., 2022) to facilitate comparisons.)

Finally, age was defined as age at first exposure—i.e., the earliest age when children attended school in a released district—and was dichotomized as early childhood (≤11 years) vs. adolescence (12+). Because having an age at first exposure required that children attended a district that was released from an integration order, our sample for examining modification by age was limited to those Black adults who as children attended a district that experienced a release before or during their school years (as opposed to Black adults who attended, as children, a district under an integration order that remained in effect through their graduation).

### 2.7 Sensitivity analyses

In sensitivity analyses, we used alternative income categories, including tertiles^1^ and contrasting the top 10%^2^ with the bottom 90% of the income distribution, providing contrasts between those living in poverty vs. those with more resources to buffer shortfalls in children's educational opportunities.

We also fit Ordinary Least Squares (OLS) models with interaction terms—i.e., regressing outcomes on school segregation, covariates, and modifiers, with interactions between a given modifier of interest and school segregation. These provided greater statistical power compared to IV estimates, though they may suffer a higher risk of confounding. They also measured a slightly different exposure, assessing not whether subgroups differ in the relationships they exhibit between health and *IV-induced resegregation* but rather between health and *school racial segregation in general*. This provides an additional test of whether, for example, the health consequences of attending more segregated schools were more severe in the South given the South's broad lack of social mobility, economic opportunity, and health resources. Put differently, our IV models estimated the health effects of increased segregation caused by court order releases (predictor = court order-induced segregation changes, outcome = health), estimated only among school districts that were under a court order during the 1990s. Our OLS estimates were also limited only to districts under a court order at that time, but instead estimated the association between segregation and health (predictor = segregation levels in general, outcome = health).

Finally, we fit several sensitivity analyses assessing whether using alternative segregation measures, or dissimilarity index values calculated using small samples, affected our results. (See Appendices H, I.)

## 3 Results

### 3.1 Sample characteristics

Most participants attended school in the South—67% of the childhood sample and 63% of the adult sample. In the adult sample, 54% were women, and among those who attended a school district that was released while they were in school, 49% were adolescents when their districts were released (Table 2).

**Table 2:** Sample distribution across strata of potential effect modifiers.

Group	*n*	*%*
**Panel A. Child sample**		
*Region*		
North	406	32.5%
South	842	67.5%
*Income*		
Below median	654	52.4%
Above median	594	47.6%
Total	1248	
**Panel B. Adult sample**		
*Region*		
North	394	37.4%
South	660	62.6%
*Income*		
Below median	530	50.3%
Above median	524	49.7%
Age at initial exposure*		
11 or below	190	50.9%
12 or above	183	49.1%
*Binary Gender*		
Men	486	46.1%
Women	568	53.9%
Total	1054	

Note: Sample sizes reflect Black participants in PSID who had complete covariate and exposure data and at least one observed outcome. Samples are smaller for age at exposure, as this sample was limited to those whose districts were ever released (as opposed to those that were ever under court order).

Covariate distributions are displayed in Table 3. Child sample participants were born, on average, in 1996, while adult sample participants were born, on average, in 1984. In both child and adult samples, baseline within-district school and residential segregation were greater in the North, where districts were also larger. Sample members largely attended high-poverty districts, with 50–67% of their districts’ students eligible for free or reduced-price lunch, depending on the region and sample.

**Table 3:** Sample characteristics, overall and by region (mean or prevalence; SD in parentheses).

A. Child health sample			
Variable	All	North	South
Baseline school segregation^a^	0.47 (0.21)	0.55 (0.21)	0.44 (0.20)
Female	48%	51%	47%
Birth year	1995.64 (7.44)	1995.25 (7.39)	1995.83 (7.47)
Baseline household income (USD)	42258.29 (38151.12)	39030.35 (42337.33)	43818 (35878.36)
Parents ever married at baseline	37%	29%	40%
**Baseline district characteristics**			
District residential segregation^a^	0.56 (0.21)	0.72 (0.17)	0.48 (0.19)
Total enrollment	95398.59 (152087.52)	174200.12 (227788.77)	57401.64 (69758.94)
% Black	49%	48%	50%
% White	31%	22%	36%
% Hispanic	16%	24%	12%
% eligible for FRPL	57%	67%	52%
**Health**			
Poor/fair/good health	35%	33%	35%
Asthma	22%	24%	21%
Obesity	9%	9%	9%
Mental/emotional problem	16%	18%	16%
Behavioral Problems Inventory^a^	8.03 (6.64)	8.35 (6.82)	7.86 (6.55)
Ever drank	38%	37%	39%
Drank at least monthly	18%	16%	20%
Heavy drinking at least monthly	5%	5%	4%
Ever smoked	33%	32%	34%
Ever smoked regularly	5%	5%	5%
Number of days smoked in last month	0.8 (4.25)	0.52 (3.12)	0.93 (4.69)
PE class >3 days/week	27%	27%	27%
Vigorous activities outside PE class >3 days/week	54%	54%	54%

B. Adult health sample			
Variable	All	North	South
Baseline school segregation^a^	0.43 (0.21)	0.48 (0.22)	0.39 (0.2)
Female	53%	54%	52%
Birth year	1984.3 (6)	1984.39 (5.76)	1984.24 (6.16)
Baseline household income *per capita*	13549.66 (13309.68)	12681.23 (13133.65)	14092.55 (13397.16)
**Baseline parental marital status**			
Married	53%	46%	58%
Single	22%	31%	17%
Separated/widowed/divorced	25%	23%	26%
**Baseline district characteristics**			
District residential segregation^a^	0.56 (0.22)	0.68 (0.2)	0.49 (0.2)
Total enrollment	103700.66 (171137.46)	176294.91 (240852.75)	58319.26 (77570.71)
% Black	44%	41%	46%
% White	35%	28%	40%
% Hispanic	17%	25%	12%
% eligible for FRPL	55%	62%	50%
**Health**			
Psychological distress^a^	4.08 (4.07)	4.39 (4.27)	3.88 (3.93)
Good health	88%	86%	89%
Current smoker	25%	28%	23%
Number of cigarettes	2.49 (5.78)	2.84 (5.92)	2.27 (5.68)
Ever drank alcohol	66%	69%	65%
Heavy drinking	22%	23%	22%
Heart disease	1%	1%	1%
Hypertension	13%	11%	14%
Diabetes	3%	3%	3%
Hours of vigorous physical activity/week	2.28 (3.26)	2.4 (3.96)	2.21 (2.74)
BMI	28.26 (6.5)	28.14 (6.02)	28.33 (6.78)

Note: Sample characteristics were calculated among Black PSID participants who were children for at least some amount of time in the post-*Dowell* era. Sample was restricted to those with complete covariate and exposure data and at least one observed outcome.

FRPL = free or reduced-price lunch; PE = physical education.

a School and district segregation were assessed via Black-White DI. Psychological distress was measured via Kessler-6 Psychological Distress Scale (continuous, 0–24). Behavior Problems Inventory range: 0–27.

### 3.2 Modification by region

In first-stage models, we tested whether F-statistics from models regressing school segregation on our IVs were sufficiently strong to support an IV analysis (Appendix B). A threshold of F > 10 is a common rule of thumb; below this, estimates from IV models are likely to be biased, due to overfitting the first stage (Staiger & Stock, 1997). F-statistics in the adult sample were well above 10 in the South (roughly 33–35, depending on the outcome)—similar to prior work (Kim et al., 2022). A 1-unit change in the IV (from spending 0% of one's observed schooling years in released districts to spending 100% of one's observed schooling years there) changed segregation levels by roughly 0.82 standard deviations (SDs). In the North, however, F-statistics were weaker (∼3–4), and the relation between our IV and school segregation levels was attenuated (0.56–0.62 SDs per unit of the IV). We found similar results for the child sample, with F-statistics >10 in the South but <10 in the North, with a slightly stronger IV-segregation relationship in the South (a change of 0.06 SDs in the DI for each additional year since dismissal) than in the North (0.05 SDs). Thus, our IV's strength was disproportionately driven by the South; inferences from IV models using the Northern sample are likely to be biased, with low statistical efficiency.

**Fig. 1:**
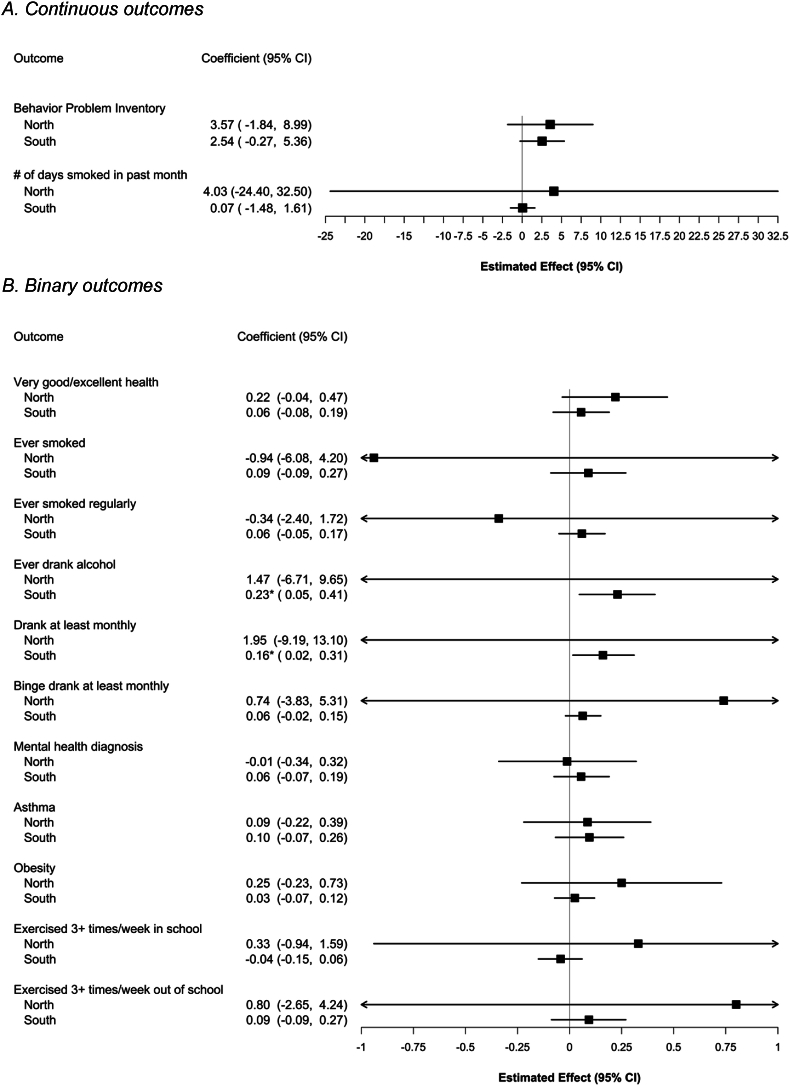
IV estimates: the impact of school racial segregation on child health, by region Note: Estimates are derived from separate IV models for each outcome, stratified by region and estimated on PSID data; they correspond to a 1-SD change in Black-White DI. The number of individuals for each model ranged from 156 to 406 in the North or 332–841 in the South, depending on outcome.

**Fig. 2:**
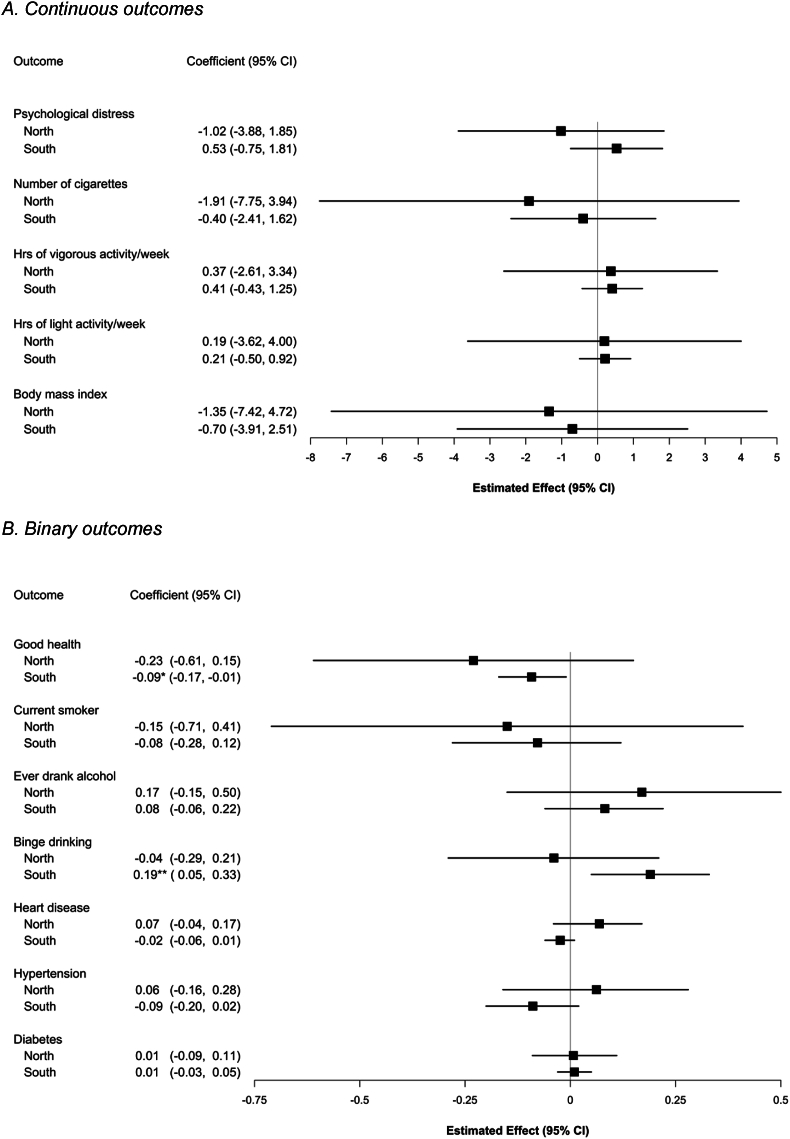
IV estimates: impact of school racial segregation on adult health, by region Note: Estimates are derived from separate IV models for each outcome, stratified by region, run on PSID data; they correspond to a 1-SD change in Black-White DI. The number of individuals for each model ranged from 271 to 393 in the North or 470–659 in the South, depending on outcome.

Full IV models also differed by region. In the South, results mirrored those from prior work that did not stratify by region (Kim et al., 2022; Wang et al., 2022). Among children (Fig. 1), findings suggested increased segregation was associated with more behavioral problems (2.54 points, 95%CI -0.27, 5.36; p = 0.076) and unhealthier drinking behaviors (ever drank: 0.23, 95%CI 0.05, 0.41; p = 0.014; drank at least monthly: 0.16, 95%CI 0.02, 0.31; p = 0.014). Among adults (Fig. 2), increasing segregation was associated with higher probability of heavy drinking (0.19 per SD of segregation, 95%CI 0.05, 0.33; p = 0.008) and lower probability of good self-reported health (−0.09, 95%CI -0.17, −0.04; p = 0.021).

In contrast, in the North, confidence intervals crossed the null for all child and adult outcomes. Confidence intervals were sufficiently wide that, in many cases, strongly positive or strongly negative effects of segregation would be consistent with our findings. Because estimates in the North were extremely imprecise, we forewent formal interaction models. We similarly forewent applying weak IV estimation methods to calculate more appropriate (wider) confidence intervals in the North (Andrews, Stock, & Sun, 2019), given that confidence intervals were already uninformatively wide in that region; we simply note that even these wide CIs underestimate our uncertainty.

### 3.3 Modification by individual characteristics

F-statistics for all individual-level subgroups were >10 (Appendix C). In second-stage models, interaction terms between all individual-level characteristics and school segregation failed to reject the null (Appendix D). Point estimates were similar and in the same direction across different groups, with no clear pattern of consistently larger effects in any group.

### 3.4 Sensitivity analyses

As sensitivity analyses, we fit OLS models regressing health on school segregation and our confounders, including an interaction between school segregation and region (Appendix E).

OLS models generated two core findings. First, OLS results mirrored the main findings from IV models. Segregation was associated with a lower probability of good health and a higher probability of heavy drinking among Black adults (statistically significantly), and more heavy drinking and a greater number of behavioral problems among Black children in the South (CIs crossed the null). As found in previous work (Kim et al., 2022; Wang et al., 2022), however, OLS models yielded estimates closer to a null or salubrious effect of segregation compared to IV models, suggesting that OLS estimates were confounded in ways that IV estimates were not (or that local average treatment effects in our IV models differed substantially from the relationship between segregation and health writ large).

However, second, broadly comparing point estimates from the North vs. the South was instructive. In region-stratified models, point estimates in the South were either closer to a more harmful effect of segregation (14 outcomes out of 24 total) or were similar to those from the North (8 outcomes); point estimates in the North were generally closer to the null or indicated *better* health in more segregated districts. For example, for adult heavy drinking, stratified models showed a 1-SD increase in segregation was associated with a higher probability of heavy drinking in the South (β = 0.06, 95%CI 0.02, 0.10, p = 0.004), but yielded a slightly negative association between segregation and heavy drinking in the North (−0.01, 95%CI -0.06, 0.04, p = 0.645).

Accordingly, interaction models indicated coefficients were statistically significantly closer to a harmful effect in the South for four outcomes—adult heavy drinking and childhood asthma, mental/emotional problems, and behavioral problems. In comparison, coefficients were statistically significantly closer to a harmful effect in the North for only two outcomes (childhood probability of ever smoking regularly or getting regular exercise outside of PE class, neither of which mirrored IV findings).

In further sensitivity analyses, alternative income categories did not shift our conclusions of no evidence for modification by income.

Finally, changing our segregation measure or dropping districts with few Black students (which could affect how meaningfully interpretable our results are when using the dissimilarity index) did not substantively alter our results (Appendices H, I).

## 4 Discussion

We examined heterogeneity in the association between school racial segregation and Black students’ health using quasi-experimental methods. We focused on school districts that were under a segregation order through the 1990s, when the Supreme Court made it much easier to be released from those orders. This allowed for more rigorous methods (leveraging quasi-randomly timed releases), and also provided a focus on the health impacts of specific legal changes enacted by federal courts.

Geographically, results show that recent quantitative evidence about school segregation's health harms may only generalize to Southern states. Southern resegregation may have driven more behavioral problems, unhealthier drinking behaviors, and lower probability of good self-reported health as Black students aged, though results for most outcomes were null. In contrast, IV estimates using Northern data yielded wide, uninformative confidence intervals.

These diverging results in the North vs. the South may reflect real regional differences in the relationships between court-ordered integration, school racial segregation, and health, but they may also reflect data limitations future research will need to address. Below, we expand on the research and policy implications of first-stage IV, OLS, and second-stage IV results.

### 4.1 Implications from first-stage IV models

First-stage models indicated that Southern districts resegregated faster after they were released from court-ordered integration plans (Reardon et al., 2012a).

This may reflect important regional differences in how segregation operates. Northern districts had higher levels of within-district segregation on average; modest increases in segregation following the end of court integration orders may thus simply have made little difference to families in districts that were already highly segregated. Relatedly, regions may have differed in *between-district* segregation, a phenomenon driven by “White flight” to the suburbs in the wake of *Brown* (Logan, Zhang, & Oakley, 2017). If White flight happened more completely in the North, changes to integration orders governing *within-district* segregation may have yielded minor changes to families’ incentive structures given the existing extremity of segregation *between* districts (Jang, 2022; John R. Logan, Oakley, & Stowell, 2008). If White flight happened less completely in the South, conversely, ending court-ordered integration within districts would have a larger impact, with potentially stronger pressure from Southern White families towards within-district resegregation.

Alternatively, Northern districts may plausibly have been more willing to voluntarily continue integration plans either without court oversight or as part of agreements to end court oversight, as in St. Louis, MO (Crouch, 2016). Future research addressing these questions is needed to guide regional integration efforts and epidemiologic measurement.

### 4.2 Implications from OLS models

A weaker IV-segregation relationship in the North, however, may not have been the sole driver of regional effect heterogeneity, as indicated by our OLS results. Specifically, OLS point estimates in the South were broadly closer to a harmful effect of segregation than they were in the North. This included interaction terms indicating segregation was associated with more adult heavy drinking in the South—but not the North—and that the estimated association between segregation and children's behavioral problems was closer to a harmful effect in the South, both of which emerged as significant effects in Southern IV models.

These regional differences could be explained by several factors. Given lower economic mobility, healthcare access, and health resources in the South (Artiga, 2016; Connor & Storper, 2020; Dollar et al., 2020; Jones, 2019; Parcha et al., 2021), the health consequences of school segregation—i.e., the health penalties faced by people with poorer educational attainment—may be more severe there. Alternatively, the distribution of educational resources in the North may not be as powerfully shaped by segregation as in the South. That is, the differential between resources available in segregated schools Black students attended in the North vs. those White students attended in the North may have been lower than the equivalent differential in the South. To our knowledge, this has rarely been tested, and existing evidence is equivocal; many Northern districts remain fiercely segregated in ways that deprive Black students of their right to quality educations (Joffe-Walt, 2020; Kozol, 1991).

Future research exploring (mediators of) school segregation's differential consequences for health across regions is necessary to inform interventions and mitigate health harms.

### 4.3 Implications from individual-level heterogeneity models

Though our age-at-first-exposure analyses were underpowered, none of our individual-level heterogeneity analyses yielded even a suggestive pattern that segregation's effects differed strongly or consistently across income, gender, or age. It is possible this is an artifact of random or limited variation in exposures (see below), but there may be substantive explanations.

For example, while higher-income families may be able to purchase more educational enrichment opportunities than poorer families, they may not disproportionately increase investments in children's educations *as segregation rises*—at least not in ways that offset segregation's health harms. This may be because A) only very expensive, rarely affordable investments would reduce the health effects of rising segregation (such as moving to a more integrated or better-resourced district) (Datar & Mason, 2008); B) *no* degree of financial investment would be health-protective (e.g., moving to a better-resourced district could have harmful effects of its own) (Keene & Padilla, 2010; Schmidt et al., 2017); or C) slow declines in educational resources as segregation rises may be difficult for parents to detect and respond to effectively. Research is needed on how contemporary parents of different incomes adaptively respond (Datar & Mason, 2008; Malik & Hagler, 2016; Northwestern Mutual, 2018) to changes in children's educational environments in the face of local increases in school segregation.

### 4.4 Limitations

This analysis has caveats. First, to be interpreted causally, IV models rely on several assumptions. While violations of these assumptions are possible, past research (Kim et al., 2022; Lutz, 2011; Orfield et al., 2016; Reardon et al., 2012a; Wang et al., 2022) offers compelling evidence that these assumptions are likely met here (section 2.6). Still, assumptions 2 and 3 cannot be affirmatively proven, an inherent limitation of IV methods. Moreover, second, our use of IV methods limits the generalizability and alters the interpretation of our findings: results can only generalize to districts that were under a court integration order through the early 1990s, and Northern and Southern districts put under integration orders may differ in ways not captured by our district-level covariates.

Third, our effect heterogeneity analyses suffer from limited variation in our modifiers. For example, we may have been unable to observe heterogeneity by income because our sample was largely low- and middle-income. A sample with more Black students from higher-income households may have yielded clearer income-based effect modification. Similarly, our sample included nearly twice as many Southern respondents as Northern ones; a larger Northern sample might add sufficient power that effects would emerge there despite a weaker IV-exposure slope. Different scales of school segregation—e.g., within schools or between districts—may also have been more salient predictors of health, which we do not capture.

Fourth, outcomes and many covariates were self-reported, potentially introducing measurement error and residual confounding.

## 5 Conclusions

This study is among the first to show that contemporary school resegregation has a particularly deleterious effect on Black health in the South—though, again, results for many outcomes were null. It does so using a rigorous, quasi-experimental design, contributing new evidence that education interventions to improve Black Southerners' well-being are urgently needed. Legislation like the Strength in Diversity Act, which would leverage federal funds to foster school integration, may have positive spillover effects on Black Southerners' health (Ayudant, 2020). The same may be true of funding formulas that would redistribute educational funding more equitably within states and districts (North, 2021). Further, education scholars point to the restoration of Black Americans' voting rights as an important precondition to winning educational racial equity (Anderson, 1988; Freeman, 1977); efforts to restore the Voting Rights Act may thus facilitate the prevention of segregation's health harms (Álvarez, 2022; Brockell, 2021; BrennanCenterfor Justice, 2022).

More broadly, our paper underscores that education policies can be health policies. Although associations for numerous outcomes were null, school segregation may impact key aspects of Black Southerners' wellness, not just their education – particularly, childhood behavioral problems, as well as long-term unhealthy drinking behaviors and self-rated health. To advance policymaking, researchers must continue to evaluate segregation's health effects across the US, with attention to segregation at multiple scales.

## Data statement

The data that support the findings of this study are available from the Panel Study of Income Dynamics (https://psidonline.isr.umich.edu/), as well as from online repositories created by Reardon et al. (2012b) and ProPublica (ProPublica, 2014). Interested researchers must apply for access to geographic data, which are not publicly available.

## Financial disclosure

This study was supported by a grant from the 10.13039/100000002National Institutes of Health (R01 HL151638). The collection of PSID data by the 10.13039/100007270University of Michigan Institute for Social Research was partly supported by the 10.13039/100000002National Institutes of Health (grant numbers R01 HD069609 and R01 AG040213) and the 10.13039/100000001National Science Foundation (award numbers SES 1157698 and 1623684). No funder had any role in the design, methods, subject recruitment, data collection, analysis, or preparation of this manuscript. The authors have no financial interests to disclose.

## Ethics statement

This study was approved by the institutional review board at University of California, San Francisco (18-25536 and 21-33615). All study activities were completed in compliance with laws on the protection of human subjects in research and related institutional and legal guidelines. At no point did the authors have access to personally identifiable information about respondents.

## CRediT authorship contribution statement

**Gabriel L. Schwartz:** Writing – review & editing, Writing – original draft, Visualization, Validation, Methodology, Formal analysis, Data curation, Conceptualization. **Guangyi Wang:** Writing – review & editing, Methodology, Formal analysis, Data curation. **Min Hee Kim:** Writing – review & editing, Methodology, Formal analysis, Data curation. **M. Maria Glymour:** Writing – review & editing, Funding acquisition, Conceptualization. **Justin S. White:** Writing – review & editing, Methodology, Funding acquisition, Conceptualization. **Daniel Collin:** Writing – review & editing, Data curation. **Rita Hamad:** Writing – review & editing, Supervision, Resources, Project administration, Methodology, Funding acquisition, Data curation, Conceptualization.

## Declaration of competing interest

None.

## Data Availability

The authors do not have permission to share data.

## Footnotes

1 Tertiles defined as: $0–19,713, $19,714-$47,439, and $47,534-$427,546 annual total household income for our child health analysis; $0–4,550, $4551-11,500, and $11,501-$94,206 annually *per capita* for our adult health analysis.

2 Top 10% defined as: >$88,500 annual total household income for our child health analysis; ∼$29,000 or more annually *per capita* for our adult health analysis.
