# Supplementary material for: Individual and regional differences in the effects of school racial segregation on Black students’ health
Source: SSM Popul Health. 2024 May 20;26:101681. doi: 10.1016/j.ssmph.2024.101681 (PMC11152755; doi:10.1016/j.ssmph.2024.101681)
Supplement: Multimedia component 1 [file mmc1.docx]

**Appendix A. Instrumental variable equations & specification**

Our instrumental variables analyses were each accomplished in two stages: a first stage regressing within-district school segregation on our IVs and covariates, and a second stage regressing our health outcomes on changes in school segregation predicted by our first stage.

*First Stage*

$seg= \alpha_{1}IV+\alpha_{2}mod+\alpha_{3}\left( IV\times mod \right)+\boldsymbol{\alpha}_{\boldsymbol{4}}^{\boldsymbol{'}}\boldsymbol{L+}\varepsilon_{1}$ (1)

$\left( seg\times mod \right)=\beta_{1}IV+\beta_{2}mod+\beta_{3}\left( IV\times mod \right)+\boldsymbol{\beta}_{\boldsymbol{4}}^{\boldsymbol{'}}\boldsymbol{L+}\varepsilon_{2}$ (2)

*Second Stage*

$y=\gamma_{1}\hat{seg}+\gamma_{2}\hat{(seg\times mod)}+\gamma_{3}mod+\boldsymbol{\gamma}_{\boldsymbol{4}}^{\boldsymbol{'}}\boldsymbol{L+}\varepsilon_{3}$ (3)

where *seg* represents levels of within-district racial school segregation, *IV* represents values of our instrumental variables, *mod* represents values of our effect modifiers, ***L*** represents a vector of a constant and our covariates, *y* represents a given outcome, and $\varepsilon$ represents “error” (individual variation around a given predicted outcome value). In Equation 1, we predict levels of school segregation with our IV, our effect modifier, an interaction between the two, and our covariates. In Equation 2, we predict levels of an interaction between school segregation and our effect modifier with the same variables. Finally, in Equation 3, we regress a given health outcome on predicted levels of segregation and the segregation $\times$ modifier interaction, our modifier, and ***L***.

Here, our IV is defined as either A) for child health, the number of years elapsed since a child’s district was released from a court order, averaged across all their childhood observations between the start of their schooling and the year a given health outcome was measured, or B), for adult health, the proportion of participants’ school-aged observations after 1991 in which a child attended a released district.

**Appendix B.** **Instrumental variables analysis first-stage results of the association between timing of court release and school racial segregation, by region and outcome for child and adult health**

*A. Child health*

| **Outcome** | **Estimate** | **Region** | |
| --- | --- | --- | --- |
|  |  | *North* | *South* |
| Poor, fair, good health | Coef. | 0.049 | 0.059 |
|  | p | 0.003 | < 0.001 |
|  | F | 9.39 | 35.93 |
| Asthma | Coef. | 0.049 | 0.059 |
|  | p | 0.003 | < 0.001 |
|  | F | 9.4 | 35.72 |
| Obesity | Coef. | 0.046 | 0.065 |
|  | p | 0.016 | < 0.001 |
|  | F | 6.17 | 39.36 |
| Mental/emotional problem | Coef. | 0.049 | 0.059 |
|  | p | 0.003 | < 0.001 |
|  | F | 9.4 | 35.15 |
| Behavior Problems Inventory | Coef. | 0.047 | 0.06 |
|  | p | 0.007 | < 0.001 |
|  | F | 7.83 | 34.11 |
| Physical education class > 3 days/week | Coef. | 0.036 | 0.15 |
|  | p | 0.481 | < 0.001 |
|  | F | 0.51 | 91.29 |
| Vigorous activities outside physical education class >3 days/week | Coef. | 0.033 | 0.15 |
|  | p | 0.529 | < 0.001 |
|  | F | 0.4 | 90.73 |
| Ever drank alcohol | Coef. | 0.029 | 0.15 |
|  | p | 0.701 | < 0.001 |
|  | F | 0.15 | 76.78 |
| Drinks alcohol at least monthly | Coef. | 0.029 | 0.14 |
|  | p | 0.701 | < 0.001 |
|  | F | 0.15 | 75.95 |
| Consumes ≥5 drinks at a time at least monthly | Coef. | 0.029 | 0.15 |
|  | p | 0.701 | < 0.001 |
|  | F | 0.15 | 76.78 |
| Ever smoked | Coef. | 0.025 | 0.15 |
|  | p | 0.729 | < 0.001 |
|  | F | 0.12 | 75.71 |
| Ever smoked regularly | Coef. | 0.025 | 0.15 |
|  | p | 0.729 | < 0.001 |
|  | F | 0.12 | 74.89 |
| No. of days smoked | Coef. | 0.025 | 0.15 |
|  | p | 0.729 | < 0.001 |
|  | F | 0.12 | 75.64 |

*B. Adult health*

| **Outcome** | **Estimate** | **Region** | |
| --- | --- | --- | --- |
|  |  | *North* | *South* |
| Self-rated health | Coef. | 0.59 | 0.82 |
|  | p | 0.075 | < 0.001 |
|  | F | 3.30 | 35.07 |
| Current smoker | Coef. | 0.59 | 0.81 |
|  | p | 0.080 | < 0.001 |
|  | F | 3.19 | 33.92 |
| # of cigarettes per day | Coef. | 0.58 | 0.81 |
|  | p | 0.079 | < 0.001 |
|  | F | 3.20 | 34.09 |
| Ever drank alcohol | Coef. | 0.58 | 0.81 |
|  | p | 0.082 | < 0.001 |
|  | F | 3.15 | 34.04 |
| Binge drinking | Coef. | 0.59 | 0.82 |
|  | p | 0.078 | < 0.001 |
|  | F | 3.22 | 34.41 |
| Psych. distress (K6) | Coef. | 0.58 | 0.81 |
|  | p | 0.083 | < 0.001 |
|  | F | 3.11 | 33.70 |
| Hours vigorous phys. activity per week | Coef. | 0.62 | 0.81 |
|  | p | 0.065 | < 0.001 |
|  | F | 3.56 | 33.32 |
| Hours light phys. activity per week | Coef. | 0.61 | 0.83 |
|  | p | 0.068 | < 0.001 |
|  | F | 3.48 | 35.45 |
| BMI | Coef. | 0.56 | 0.82 |
|  | p | 0.096 | < 0.001 |
|  | F | 2.86 | 35.29 |
| Heart disease | Coef. | 0.58 | 0.81 |
|  | p | 0.082 | < 0.001 |
|  | F | 3.15 | 34.07 |
| Hypertension | Coef. | 0.58 | 0.81 |
|  | p | 0.082 | < 0.001 |
|  | F | 3.14 | 34.02 |
| Diabetes | Coef. | 0.58 | 0.81 |
|  | p | 0.082 | < 0.001 |
|  | F | 3.15 | 34.02 |

**Appendix C.** **Instrumental variables first-stage results (F-statistics) for the association between court order releases and school racial segregation, by individual-level modifier, outcome, and life stage**

| **Life stage** | **Health outcome** | **Gender** | | **Childhood Household Income** | | **Age at first exposure** | |
| --- | --- | --- | --- | --- | --- | --- | --- |
|  |  | *Men* | *Women* | *Low* | *High* | *Young* | *Old* |
| **Adults** | Psychological distress (K6) | 23.89 | 31.40 | 19.19 | 30.99 | 70.52 | 16.18 |
|  | Good health | 26.07 | 33.22 | 22.44 | 30.77 | 69.43 | 19.72 |
|  | Current smoker | 25.62 | 32.23 | 21.31 | 29.93 | 70.76 | 20.18 |
|  | Number of cigarettes | 25.63 | 32.55 | 21.23 | 30.83 | 78.57 | 19.61 |
|  | Ever drank alcohol | 25.62 | 32.36 | 21.15 | 30.28 | 69.43 | 20.01 |
|  | Binge drinking | 26.13 | 33.11 | 22.10 | 30.28 | 69.16 | 20.18 |
|  | Heart disease | 25.64 | 32.41 | 21.08 | 30.29 | 69.43 | 19.74 |
|  | Hypertension | 25.56 | 32.40 | 21.07 | 30.28 | 69.43 | 19.74 |
|  | Diabetes | 25.56 | 32.40 | 21.06 | 30.28 | 69.43 | 19.74 |
|  | Vigorous physical activity (hrs/week) | 24.91 | 33.98 | 20.96 | 31.20 | 71.69 | 21.69 |
|  | BMI | 25.39 | 34.09 | 21.85 | 30.16 | 66.40 | 19.50 |
|  |  |  |  |  |  |  |  |
| **Children** | Poor/fair/good health |  |  | 27.55 | 56.64 |  |  |
|  | Asthma |  |  | 27.28 | 56.94 |  |  |
|  | Obesity |  |  | 23.33 | 64.58 |  |  |
|  | Mental/emotional problem |  |  | 27.41 | 55.16 |  |  |
|  | Behavioral Problems Inventory |  |  | 25.87 | 52.08 |  |  |
|  | Ever drank |  |  | 31.93 | 39.59 |  |  |
|  | Drank at least monthly |  |  | 31.93 | 37.57 |  |  |
|  | Binged on alcohol at least monthly |  |  | 31.93 | 39.59 |  |  |
|  | Ever smoked |  |  | 31.89 | 36.48 |  |  |
|  | Ever smoked regularly |  |  | 31.89 | 36.02 |  |  |
|  | Number of days smoked in the last month |  |  | 31.89 | 36.57 |  |  |
|  | PE class > 3 days/week |  |  | 36.76 | 59.10 |  |  |
|  | Vigorous phys. act, outside PE class >3 days/week |  |  | 35.70 | 59.20 |  |  |

**Appendix D.** **Instrumental variables analysis estimates of the effect of school segregation on health, by individual-level modifier levels and life stage**

| **Modifier** | **Life Stage** | **Outcome** | **Coef.** | **95% CI** | **p** | **Coef.** | **95% CI** | **p** | **Interaction p** |
| --- | --- | --- | --- | --- | --- | --- | --- | --- | --- |
|  |  |  | *Young* | | | *Old* | | |  |
| *Age* | **Adult** | Psychological distress (K6) | 4.43 | (-3.57 - 12.44) | 0.278 | 3.25 | (-19.46 - 25.97) | 0.779 | 0.586 |
|  |  | Good health | -0.59* | (-1.05 - -0.13) | 0.012 | -1.68* | (-3.16 - -0.20) | 0.026 | 0.667 |
|  |  | Current smoker | -0.57 | (-1.44 - 0.30) | 0.202 | 0.90 | (-1.54 - 3.34) | 0.469 | 0.316 |
|  |  | Number of cigarettes | -4.91 | (-11.88 - 2.07) | 0.168 | 7.44 | (-17.72 - 32.61) | 0.562 | 0.642 |
|  |  | Ever drank alcohol | 0.04 | (-0.88 - 0.96) | 0.935 | -0.01 | (-1.76 - 1.73) | 0.989 | 0.833 |
|  |  | Binge drinking | 1.17** | (0.37 - 1.98) | 0.004 | 1.29+ | (-0.15 - 2.72) | 0.079 | 0.865 |
|  |  | Heart disease | -0.05 | (-0.11 - 0.02) | 0.157 | 0.10 | (-0.10 - 0.31) | 0.329 | 0.377 |
|  |  | Hypertension | -0.15 | (-0.65 - 0.36) | 0.568 | -0.12 | (-1.33 - 1.08) | 0.840 | 0.764 |
|  |  | Diabetes | -0.10 | (-0.28 - 0.08) | 0.274 | 0.18 | (-0.31 - 0.66) | 0.475 | 0.511 |
|  |  | Vigorous physical activity (hrs/week) | 3.78 | (-1.11 - 8.66) | 0.129 | -0.46 | (-11.42 - 10.50) | 0.934 | 0.483 |
|  |  | BMI | 13.32* | (0.06 - 26.59) | 0.049 | -1.83 | (-37.29 - 33.62) | 0.919 | 0.128 |
|  |  |  | *Men* | | | *Women* | | |  |
| *Gender* | **Adult** | Psychological distress (K6) | 0.35 | (-1.05 - 1.75) | 0.624 | 0.06 | (-1.75 - 1.88) | 0.945 | 0.433 |
|  |  | Good health | -0.11 | (-0.24 - 0.03) | 0.117 | -0.08 | (-0.19 - 0.02) | 0.127 | 0.429 |
|  |  | Current smoker | -0.10 | (-0.48 - 0.28) | 0.611 | -0.10 | (-0.27 - 0.08) | 0.278 | 0.575 |
|  |  | Number of cigarettes | -0.18 | (-4.84 - 4.48) | 0.940 | -1.56 | (-3.56 - 0.43) | 0.125 | 0.671 |
|  |  | Ever drank alcohol | -0.01 | (-0.22 - 0.20) | 0.944 | 0.14+ | (-0.01 - 0.28) | 0.060 | 0.264 |
|  |  | Binge drinking | 0.23* | (0.02 - 0.45) | 0.035 | 0.11+ | (-0.01 - 0.23) | 0.063 | 0.739 |
|  |  | Heart disease | -0.01 | (-0.04 - 0.02) | 0.562 | -0.01 | (-0.06 - 0.03) | 0.533 | 0.708 |
|  |  | Hypertension | -0.08 | (-0.20 - 0.04) | 0.200 | -0.09 | (-0.23 - 0.05) | 0.209 | 0.452 |
|  |  | Diabetes | 0.00 | (-0.06 - 0.07) | 0.957 | -0.00 | (-0.04 - 0.03) | 0.927 | 0.386 |
|  |  | Vigorous physical activity (hrs/week) | 0.64 | (-0.97 - 2.25) | 0.439 | 0.38 | (-0.44 - 1.21) | 0.365 | 0.341 |
|  |  | BMI | 0.10 | (-2.85 - 3.04) | 0.949 | -0.06 | (-3.50 - 3.37) | 0.971 | 0.942 |
|  |  |  | *Below Median Income* | | | *Above Median Income* | | |  |
| *Income* | **Adult** | Psychological distress (K6) | -0.37 | (-2.70 - 1.96) | 0.757 | 0.15 | (-1.17 - 1.47) | 0.829 | 0.969 |
|  |  | Good health | -0.11 | (-0.25 - 0.04) | 0.149 | -0.10* | (-0.19 - -0.00) | 0.039 | 0.308 |
|  |  | Current smoker | -0.08 | (-0.51 - 0.36) | 0.736 | -0.13 | (-0.34 - 0.07) | 0.204 | 0.901 |
|  |  | Number of cigarettes | -0.38 | (-4.65 - 3.90) | 0.862 | -1.02 | (-3.26 - 1.22) | 0.374 | 0.245 |
|  |  | Ever drank alcohol | 0.24+ | (-0.04 - 0.53) | 0.090 | 0.01 | (-0.14 - 0.16) | 0.928 | 0.318 |
|  |  | Binge drinking | 0.16+ | (-0.01 - 0.32) | 0.060 | 0.17* | (0.03 - 0.31) | 0.020 | 0.341 |
|  |  | Heart disease | -0.03 | (-0.09 - 0.03) | 0.355 | 0.02 | (-0.01 - 0.04) | 0.160 | 0.458 |
|  |  | Hypertension | -0.05 | (-0.24 - 0.13) | 0.567 | -0.07 | (-0.20 - 0.05) | 0.246 | 0.238 |
|  |  | Diabetes | -0.00 | (-0.06 - 0.05) | 0.874 | -0.00 | (-0.03 - 0.03) | 0.758 | 0.461 |
|  |  | Vigorous physical activity (hrs/week) | 0.10 | (-1.09 - 1.29) | 0.871 | 0.81+ | (-0.10 - 1.72) | 0.082 | 0.222 |
|  |  | BMI | -1.39 | (-5.39 - 2.62) | 0.498 | 0.22 | (-2.77 - 3.21) | 0.883 | 0.192 |
|  | **Child** | Poor/fair/good health | -0.02 | (-0.16 - 0.12) | 0.787 | 0.20* | (0.014 - 0.38) | 0.035 | 0.117 |
|  |  | Asthma | 0.09 | (-0.06 - 0.23) | 0.239 | 0.070 | (-0.15 - 0.28) | 0.523 | 0.769 |
|  |  | Obesity | -0.02 | (-0.13 - 0.08) | 0.658 | 0.13 | (-0.032 - 0.29) | 0.115 | 0.064 |
|  |  | Mental/emotional problem | 0.03 | (-0.08 - 0.14) | 0.599 | 0.078 | (-0.12 - 0.28) | 0.438 | 0.621 |
|  |  | Behavioral Problems Inventory | 2.60+ | (-0.15 - 5.35) | 0.064 | 2.01 | (-0.81 - 4.83) | 0.161 | 0.423 |
|  |  | Ever drank | 0.26+ | (-0.02 - 0.53) | 0.066 | 0.27* | (0.0030 - 0.54) | 0.048 | 0.123 |
|  |  | Drank at least monthly | 0.25 | (-0.05 - 0.55) | 0.101 | 0.17 | (-0.11 - 0.44) | 0.228 | 0.769 |
|  |  | Binged on alcohol at least monthly | 0.06 | (-0.06 - 0.18) | 0.336 | 0.083+ | (-0.015 - 0.18) | 0.097 | 0.396 |
|  |  | Ever smoked | 0.02 | (-0.21 - 0.25) | 0.850 | 0.038 | (-0.19 - 0.27) | 0.743 | 0.536 |
|  |  | Ever smoked regularly | 0.08 | (-0.07 - 0.23) | 0.291 | 0.015 | (-0.093 - 0.12) | 0.785 | 0.229 |
|  |  | Number of days smoked in the last month | 0.33 | (-1.23 - 1.90) | 0.671 | 1.22 | (-1.63 - 4.08) | 0.397 | 0.908 |
|  |  | PE class > 3 days/week | -0.01 | (-0.17 - 0.14) | 0.859 | 0.038 | (-0.17 - 0.25) | 0.721 | 0.761 |
|  |  | Vigorous phys. activity outside PE class >3 days/week | 0.23* | (0.06 - 0.41) | 0.011 | 0.032 | (-0.23 - 0.30) | 0.809 | 0.472 |

**Appendix E.** **OLS estimates of the association between school segregation and health, by region, and life stage**

| **Life Stage** | **Outcome** | **North** | | | **South** | | | **Interaction p** | **Point estimate closer to a harmful association in:*** |
| --- | --- | --- | --- | --- | --- | --- | --- | --- | --- |
|  |  | **Coef** | **CI** | **p** | **Coef** | **CI** | **p** |  |  |
| Child | Poor/fair/good health | -0.03 | (-0.11 - 0.04) | 0.410 | -0.02 | (-0.06 - 0.03) | 0.430 | 0.540 | Same |
|  | Asthma | -0.08* | (-0.15 - -0.01) | 0.021 | 0.03 | (-0.02 - 0.08) | 0.220 | 0.001 | South |
|  | Obesity | > -0.01 | (-0.07 - 0.07) | 0.970 | 0.01 | (-0.02 - 0.05) | 0.450 | 0.540 | Same |
|  | Mental/emotional problem | -0.04 | (-0.12 - 0.04) | 0.300 | -0.01 | (-0.05 - 0.03) | 0.630 | 0.034 | South |
|  | Behavioral Problems Inventory | -1.02+ | (-2.13 - 0.08) | 0.069 | 0.11 | (-0.62 - 0.83) | 0.770 | 0.019 | South |
|  | Ever drank | -0.01 | (-0.21 - 0.19) | 0.940 | 0.08+ | (>-0.01 - 0.17) | 0.063 | 0.530 | South |
|  | Drank at least monthly | -0.12 | (-0.31 - 0.07) | 0.210 | 0.06+ | (>-0.01 - 0.12) | 0.069 | 0.160 | South |
|  | Binged on alcohol at least monthly | -0.05 | (-0.21 - 0.12) | 0.570 | 0.05 | (-0.02 - 0.11) | 0.130 | 0.510 | South |
|  | Ever smoked | -0.08 | (-0.23 - 0.06) | 0.240 | 0.04 | (-0.05 - 0.12) | 0.390 | 0.980 | South |
|  | Ever smoked regularly | 0.06+ | >-0.01 - 0.11) | 0.052 | 0.02 | (-0.02 - 0.07) | 0.270 | 0.048 | North |
|  | Number of days smoked in the last month | 0.24 | (-0.26 - 0.73) | 0.340 | -0.02 | (-0.92 - 0.89) | 0.970 | 0.051 | South |
|  | PE class > 3 days/week | 0.05 | (-0.07 - 0.16) | 0.410 | -0.02 | (-0.08 - 0.04) | 0.510 | 0.850 | South |
|  | Vigorous physical activity outside PE class > 3 days/week | -0.10+ | (-0.21 - < 0.01) | 0.054 | 0.08* | (0.02 - 0.14) | 0.016 | < 0.001 | North |
| Adult | Psychological distress (K6) | 0.42 | (-0.17 - 1.02) | 0.157 | 0.42+ | (-0.02 - 0.85) | 0.061 | 0.567 | Same |
|  | Good health | -0.07** | (-0.12 - -0.02) | 0.004 | -0.06** | (-0.09 - -0.02) | 0.001 | 0.306 | Same |
|  | Current smoker | -0.04 | (-0.10 - 0.02) | 0.216 | 0.03 | (-0.02 - 0.08) | 0.283 | 0.250 | South |
|  | Number of cigarettes | -0.38 | (-0.88 - 0.13) | 0.140 | 0.13 | (-0.44 - 0.70) | 0.650 | 0.268 | South |
|  | Ever drank alcohol | -0.01 | (-0.09 - 0.07) | 0.805 | < 0.01 | (-0.06 - 0.06) | 0.979 | 0.256 | Same |
|  | Binge drinking | -0.01 | (-0.06 - 0.04) | 0.645 | 0.06** | (0.02 - 0.10) | 0.004 | 0.007 | South |
|  | Heart disease | 0.01 | (>-0.01 - 0.02) | 0.116 | 0.01 | (>-0.01 - 0.02) | 0.140 | 0.536 | Same |
|  | Hypertension | < 0.01 | (-0.07 - 0.07) | 0.982 | -0.01 | (-0.06 - 0.04) | 0.657 | 0.440 | Same |
|  | Diabetes | 0.02 | (-0.02 - 0.07) | 0.290 | > -0.01 | (-0.02 - 0.02) | 0.806 | 0.090 | Same |
|  | Vigorous physical activity (hrs/week) | 0.38 | (-0.09 - 0.85) | 0.112 | 0.02 | (-0.21 - 0.25) | 0.851 | 0.327 | South |
|  | BMI | 0.76 | (-0.37 - 1.90) | 0.184 | 0.83 | (-0.36 - 2.02) | 0.169 | 0.400 | South |

* "Similar" estimates are those where estimates were within 2 percentage points of one another across region.

**Appendix F. Outcome missing-ness rates (among occasions in which observed sample members were eligible to be asked each question)**

| **Life stage** | **Outcome** | **Missingness Rate** |
| --- | --- | --- |
| Childhood | Poor, fair, good health | 0.48% |
|  | Asthma | 0.42% |
|  | Obesity | 23.42% |
|  | Mental/emotional problems | 1.54% |
|  | Behavior Problems Inventory | 5.35% |
|  | Ever drank | 9.90% |
|  | Drank at least monthly | 10.07% |
|  | Binged on alcohol at least monthly | 9.90% |
|  | Ever smoked | 9.55% |
|  | Ever smoked regularly | 9.74% |
|  | Number of days smoked in the last month | 9.72% |
|  | PE class > 3 days/week | 9.56% |
|  | Vigorous physical activity outside PE class > 3 days/week | 9.42% |
| Adulthood | Psychological distrress (K6) | 3.02% |
|  | Good self-rated health | 0.28% |
|  | Smoking | 0.54% |
|  | Number of Cigarette | 0.63% |
|  | Ever drank alcohol | 0.33% |
|  | Binge drinking | 1.71% |
|  | Heart disease | 0.26% |
|  | Hypertension | 0.30% |
|  | Diabetes | 0.30% |
|  | Vigorous physical activity (hours/week) | 10.85% |
|  | BMI | 1.17% |

**Appendix G. Sample selection flow charts**

*A. Child health sample selection flowchart*

*B. Adult health sample selection flowchart*

**Appendix H. Sensitivity Analysis: Accounting for the behavior of the dissimilarity index when districts have low Black enrollments**

One limitation of the dissimilarity index is that its values are sensitive to small changes in enrollment when the number of Black (or White) students is extremely low. For example, in a district with 2 Black students and 2 schools, a single Black student moving schools could change the dissimilarity index from 1 to 0.

To assess whether this kind of low enrollment problem might impact our results, we first examined distributions of the proportion of sample members’ school districts that were Black. Cases in which there were few enough Black or White students in sample members’ districts to cause concern were very rare. In the child sample (Table A), 90% of child observations occurred in districts that were between 13% and 82% Black. Indeed, only in 2 observations (across thousands) was the percent Black < 5%; and even the 99^th^ percentile was still comprised of 3% non-Black students. Similarly in our adult sample (Table B), 90% of adults first attended school in districts that were between 6% and 83% Black.

Still, to assess whether even these rare cases might have biased our results, we re-ran our region-stratified IV models after dropping the bottom 5% of the sample in terms of the percent of students in sample members’ districts who were Black. Results were slightly less efficiently estimated, but results were effectively unchanged (Tables C and D).

*A. Child sample (across child observations)*

| **Percentile** | **Value (proportion Black)** |
| --- | --- |
| 1% | 0.95 |
| 5% | 0.13 |
| 10% | 0.19 |
| 25% | 0.37 |
| 50% (median) | 0.49 |
| 75% | 0.61 |
| 90% | 0.75 |
| 95% | 0.82 |
| 99% | 0.97 |
| *Mean (SD)* | 0.49 (0.21) |

4 Smallest Values (largest to smallest): 0.054, 0.052, 0.046, <0.01

4 Largest Values (smallest to largest): 0.995, 0.995, 0.995, 0.998

*B. Adult sample (across people, using their earliest childhood observation)*

| **Percentile** | **Value (proportion Black)** |
| --- | --- |
| 1% | 0.01 |
| 5% | 0.06 |
| 10% | 0.10 |
| 25% | 0.23 |
| 50% (median) | 0.44 |
| 75% | 0.58 |
| 90% | 0.76 |
| 95% | 0.83 |
| 99% | 0.97 |
| *Mean (SD)* | 0.44 (0.24) |

4 Smallest Values (largest to smallest): 0.003, 0.001, 0.001, 0.001

4 Largest Values (smallest to largest): 0.987, 0.987, 0.987, 0.995

*C. Child sample*

| **Outcome** | **North** | | | **South** | | |
| --- | --- | --- | --- | --- | --- | --- |
|  | *Estimate* | *95% CI* | *p* | *Estimate* | *95% CI* | *p* |
| Poor/fair/good health | 0.22+ | (-0.03 - 0.48) | 0.088 | 0.05 | (-0.08 - 0.19) | 0.429 |
| Asthma | 0.09 | (-0.22 - 0.40) | 0.554 | 0.10 | (-0.07 - 0.26) | 0.249 |
| Obesity | 0.25 | (-0.23 - 0.73) | 0.299 | 0.02 | (-0.07 - 0.12) | 0.612 |
| Mental/emotional problem | -0.02 | (-0.35 - 0.32) | 0.918 | 0.06 | (-0.08 - 0.19) | 0.401 |
| Behavioral Problems Inventory* | 3.63 | (-1.91 - 9.16) | 0.195 | 2.56+ | (-0.27 - 5.39) | 0.076 |
| Ever drank | 1.47 | (-6.71 - 9.65) | 0.717 | 0.23* | (0.05 - 0.41) | 0.014 |
| Drank at least monthly | 1.95 | (-9.19 - 13.10) | 0.723 | 0.16* | (0.02 - 0.31) | 0.03 |
| Heavy drinking at least monthly | 0.74 | (-3.83 - 5.31) | 0.743 | 0.06 | (-0.02 - 0.15) | 0.134 |
| Ever smoked | -0.94 | (-6.09 - 4.20) | 0.712 | 0.09 | (-0.09 - 0.27) | 0.323 |
| Ever smoked regularly | -0.34 | (-2.40 - 1.72) | 0.74 | 0.06 | (-0.05 - 0.17) | 0.29 |
| Number of days smoked in last month | 4.03 | (-24.41 - 32.46) | 0.775 | 0.07 | (-1.48 - 1.61) | 0.934 |
| PE class > 3 days/week | 0.33 | (-0.94 - 1.59) | 0.605 | -0.04 | (-0.15 - 0.06) | 0.413 |
| Vigorous activities outside PE class > 3 days/week | 0.80 | (-2.65 - 4.24) | 0.641 | 0.09 | (-0.09 - 0.27) | 0.307 |

*D. Adult sample*

| **Outcome** | **North** | | | **South** | | |
| --- | --- | --- | --- | --- | --- | --- |
|  | *Estimate* | *95% CI* | *p* | *Estimate* | *95% CI* | *p* |
| Psychological distress* | -1.00 | (-3.86 - 1.85) | 0.49 | 0.61 | (-0.61 - 1.83) | 0.33 |
| Good health | -0.17 | (-0.48 - 0.13) | 0.27 | -0.09* | (-0.17 - -0.01) | 0.022 |
| Current smoker | -0.09 | (-0.63 - 0.4) | 0.73 | -0.08 | (-0.27 - 0.12) | 0.45 |
| Number of cigarettes | -1.53 | (-7.35 - 4.28) | 0.61 | -0.38 | (-2.36 - 1.60) | 0.70 |
| Ever drank alcohol | 0.18 | (-0.14 - 0.50) | 0.26 | 0.08 | (-0.06 - 0.22) | 0.26 |
| Heavy drinking | < 0.01 | (-0.25 - 0.26) | 0.99 | 0.19** | (0.05 - 0.33) | 0.008 |
| Heart disease | 0.07 | (-0.04 - 0.18) | 0.19 | -0.02 | (-0.06 - 0.01) | 0.19 |
| Hypertension | 0.07 | (-0.15 - 0.29) | 0.53 | -0.09 | (-0.20 - 0.02) | 0.12 |
| Diabetes | < 0.01 | (-0.10 - 0.10) | 1.00 | 0.01 | (-0.03 - 0.05) | 0.61 |
| Hours of vigorous physical activity/week | 0.35 | (-2.59 - 3.28) | 0.82 | 0.43 | (-0.41 - 1.26) | 0.32 |

*Note:* ** p<0.01; * p<0.05; + p<0.10

**Appendix I. Sensitivity Analysis: Alternative segregation measures**

To assess whether our results were consistent when using other measures of segregation, we re-ran our region-stratified IV models, this time using the Black Isolation Index as our exposure. The isolation index is calculated as the probability that a random peer in a Black student’s school is also Black. While less directly targeted by court orders than the dissimilarity index, repeating our analyses using the isolation index helps facilitate comparisons across studies and demonstrates the importance of researchers’ choice of segregation measure.

Results using the isolation index were substantively consistent with our main findings, though associations between school segregation and behavioral problems in the South ceased to be statistically significant in this specification.

| *A. Child Sample* |  |  |  |  |  |  |
| --- | --- | --- | --- | --- | --- | --- |
|  |  |  |  |  |  |  |
| **Outcome** | **North** | | | **South** | | |
|  | *Estimate* | *95% CI* | *p* | *Estimate* | *95% CI* | *p* |
| Poor/fair/good health | 0.49+ | (-0.06 - 1.05) | 0.082 | 0.27 | (-0.40 - 0.94) | 0.431 |
| Asthma | 0.20 | (-0.45 - 0.84) | 0.543 | 0.46 | (-0.41 - 1.33) | 0.296 |
| Obesity | 0.47 | (-0.24 - 1.18) | 0.186 | 0.11 | (-0.32 - 0.53) | 0.620 |
| Mental/emotional problem | -0.03 | (-0.78 - 0.73) | 0.943 | 0.26 | (-0.39 - 0.92) | 0.428 |
| Behavioral Problems Inventory* | 8.46 | (-3.55 - 20.5) | 0.164 | 12.14 | (-4.04 - 28.33) | 0.140 |
| Ever drank | -1.27 | (-4.34 - 1.80) | 0.407 | 0.77* | (0.01 - 1.52) | 0.048 |
| Drank at least monthly | -1.69 | (-4.86 - 1.49) | 0.287 | 0.55+ | (-0.05 - 1.16) | 0.071 |
| Heavy drinking at least monthly | -0.64 | (-2.10 - 0.82) | 0.38 | 0.21 | (-0.09 - 0.52) | 0.170 |
| Ever smoked | 0.75 | (-1.72 - 3.21) | 0.541 | 0.30 | (-0.32 - 0.92) | 0.334 |
| Ever smoked regularly | 0.27 | (-0.45 - 0.99) | 0.454 | 0.20 | (-0.17 - 0.56) | 0.288 |
| Number of days smoked in last month | -3.20 | (-14.97 - 8.58) | 0.584 | 0.22 | (-4.98 - 5.42) | 0.934 |
| PE class > 3 days/week | -0.64 | (-3.55 - 2.26) | 0.656 | -0.14 | (-0.48 - 0.19) | 0.400 |
| Vigorous activities outside PE class > 3 days/week | -1.22 | (-3.91 - 1.47) | 0.364 | 0.31 | (-0.31 - 0.93) | 0.323 |
|  |  |  |  |  |  |  |

| *B. Adult Sample* |  |  |  |  |  |  |
| --- | --- | --- | --- | --- | --- | --- |
|  |  |  |  |  |  |  |
| **Outcome** | **North** | | | **South** | | |
|  | *Estimate* | *95% CI* | *p* | *Estimate* | *95% CI* | *p* |
| Psychological distress* | -2.33 | (-10.00 - 5.38) | 0.55 | 1.00 | (-1.57 - 3.58) | 0.45 |
| Good health | -0.52 | (-1.28 - 0.24) | 0.18 | -0.18* | (-0.34 - -0.01) | 0.038 |
| Current smoker | -0.35 | (-1.81 - 1.10) | 0.63 | -0.15 | (-0.51 - 0.22) | 0.44 |
| Number of cigarettes | -4.41 | (-20.3 - 11.4) | 0.59 | -0.75 | (-4.50 - 3.00) | 0.69 |
| Ever drank alcohol | 0.40 | (-0.38 - 1.18) | 0.32 | 0.16 | [-0.14 - 0.45] | 0.3 |
| Heavy drinking | -0.09 | (-0.69 - 0.52) | 0.78 | 0.35* | (0.06 - 0.65) | 0.018 |
| Heart disease | 0.16 | (-0.08 - 0.39) | 0.19 | -0.05 | (-0.11 - 0.02) | 0.17 |
| Hypertension | 0.14 | (-0.33 - 0.61) | 0.55 | -0.17+ | (-0.36 - 0.03) | 0.088 |
| Diabetes | 0.02 | (-0.21 - 0.25) | 0.89 | 0.02 | (-0.05 - 0.09) | 0.61 |
| Hours of vigorous physical activity/week | 0.83 | (-5.99 - 7.64) | 0.81 | 0.77 | (-0.78 - 2.32) | 0.33 |

*Note:* ** p<0.01; * p<0.05; + p<0.10
